# Vendor‐independent skin dose mapping application for interventional radiology and cardiology

**DOI:** 10.1002/acm2.13167

**Published:** 2021-01-13

**Authors:** Marko Krajinović, Nikola Kržanović, Olivera Ciraj‐Bjelac

**Affiliations:** ^1^ School of Electrical Engineering University of Belgrade Belgrade Serbia; ^2^ „VINČA" Institute of Nuclear Sciences ‐ National Institute of the Republic of Serbia University of Belgrade Belgrade Serbia

**Keywords:** DICOM RDSR, interventional procedures, skin dose assessment, SkinCare application, software, XR‐RV3 GafChromic films

## Abstract

**Purpose:**

The purpose of this paper is to present and validate an originally developed application SkinCare used for skin dose mapping in interventional procedures, which are associated with relatively high radiation doses to the patient’s skin and possible skin reactions.

**Methods:**

SkinCare is an application tool for generating skin dose maps following interventional radiology and cardiology procedures using the realistic 3D patient models. Skin dose is calculated using data from Digital Imaging and Communications in Medicine (DICOM) Radiation Dose Structured Reports (RDSRs). SkinCare validation was performed by using the data from the Siemens Artis Zee Biplane fluoroscopy system and conducting “Acceptance and quality control protocols for skin dose calculating software solutions in interventional cardiology” developed and tested in the frame of the VERIDIC project. XR‐RV3 Gafchromic films were used as dosimeters to compare peak skin doses (PSDs) and dose maps obtained through measurements and calculations. DICOM RDSRs from four fluoroscopy systems of different vendors (Canon, GE, Philips, and Siemens) were used for the development of the SkinCare and for the comparison of skin dose maps generated using SkinCare to skin dose maps generated by different commercial software tools (Dose Tracking System (DTS) from Canon, RadimetricsTM from Bayer and RDM from MEDSQUARE). The same RDSRs generated during a cardiology clinical procedure (percutaneous coronary intervention—PCI) were used for comparison.

**Results:**

Validation performed using VERIDIC's protocols for skin dose calculation software showed that PSD calculated by SkinCare is within 17% and 16% accuracy compared to measurements using XR‐RV3 Gafchromic films for fundamental irradiation setups and simplified clinical procedures, respectively. Good visual agreement between dose maps generated by SkinCare and DTS, Radimetrics^TM^ and RDM was obtained.

**Conclusions:**

SkinCare is proved to be very convenient solution that can be used for monitoring delivered dose following interventional procedures.

## 1 INTRODUCTION

Interventional procedures in radiology and cardiology are associated with relatively high radiation doses to the patient’s skin which may lead to skin reactions.^1^ Even though peak skin dose (PSD) assessment can be accomplished with a wide selection of detectors,^2^ using software tools is more convenient and economical. At the present time, most equipment vendors have developed online solutions for skin dose calculations. CareMonitor by Siemens and DoseWise by Philips are solutions which basically provide the accumulated peak air kerma in the current projection.^3^ On the other hand, Dose Map from GE is an advanced two‐dimensional (2D) solution,^4^, ^5^, ^6^ while Dose Tracking System (DTS) from Canon (Toshiba) represents a state of the art three‐dimensional (3D) solution for skin dose mapping.^7^, ^8^, ^9^ The most important drawback of previously mentioned solutions is that they are vendor specific and cannot be used in fluoroscopy systems from other manufacturers.

Utilizing Digital Imaging and Communications in Medicine (DICOM) Radiation Dose Structured Report (RDSR) generated at the end of the intervention is the only way to make vendor‐independent solutions.^10^ RDSR was added to the DICOM standard^11^ with intention to standardized format of recording all the information related to the exposure parameters used for each irradiation event undergone by the patient. RDSR contains all the necessary technical, geometric, and dosimetric data necessary to assess the patient skin dose. In addition to online solutions mentioned above, there are commercial offline software tools which utilize DICOM headers and/or RDSRs for skin dose calculations such as em.dose from Esprimed,^12^, ^13^ RDM by MEDSQUARE,^3^ DOSE from Qaelum,^14^ NEXO[DOSE]® by Bracco,^15^, ^16^ Radimetrics^TM^ from Bayer,^17^ and Skin Dose Map® tool integrated in DoseWatch® by GE Healthcare.^18^ Other software solutions can be found in literature.^19^, ^20^, ^21^, ^22^, ^23^

The objective of this paper is to present an originally developed skin dose mapping application SkinCare that can be readily used with interventional units from different manufacturers. The application was validated by using the data from the Siemens Artis Zee fluoroscopy system and conducting the “Acceptance and quality control protocols for skin dose calculating software solutions in interventional cardiology” developed and tested in the frame of the Validation and Estimation of Radiation Skin Dose in Interventional Cardiology (VERIDIC) project.^24^ VERIDIC project, funded under European Joint Programme for the Integration of Radiation Protection Research, H2020 (Grant agreement No 662287), was focused on the skin dose calculation (SDC) software products in interventional cardiology, with an aim to contribute to the harmonization and the validation of SDC software products in interventional cardiology.^24^ Additionally, SkinCare’s dose maps generated using different RDSRs were compared visually with different validated commercial software tools for Canon (Toshiba), GE, Philips, and Siemens fluoroscopy systems in order to verify the correctness of applied geometric algorithm.

## 2 MATERIALS AND METHODS

### 2.A SkinCare

SkinCare is an application tool for generating skin dose maps following interventional radiology and interventional cardiology procedures using the realistic 3D patient models. RDSRs from Canon (Toshiba), GE, Philips, and Siemens were used for development of the application, making SkinCare compatible with major fluoroscopy unit vendors. SkinCare is a standalone desktop application that runs in any of the available web browsers. Easy configuration of the system correction factors and patient models provide fast way for generating skin dose maps and visualization of RDSR content.

#### 2.A.1 Patient modeling

Patient models were created using MB‐Lab,^25^ an open‐source plug‐in for free and open‐source 3D computer graphics software Blender.^26^ SkinCare has a library of 42 3D patient models of height ranging from 150 cm to 210 cm, with an increment of 10 cm. Male and female models consider three different body types: thin, standard size, and obese. All models have arms‐down pose corresponding to patient supine position. Two additional models of sizes 30 × 30 × 15 cm^3^ and 35.56 cm × 43.52 cm (14" × 17") represent water phantom and XR‐RV3 Gafchromic film, respectively, for the purpose of quality control (QC) tests.

#### 2.A.2 Patient positioning

Since different manufacturers of the fluoroscopy systems define 3D position of isocenter in relation to proprietary point in space, it is necessary to determine the offsets for RDSR’s attributes *Table Lateral Position*, *Table Longitudinal Position,* and *Table Height Position*. This can be done by positioning the surface of table head end at the isocenter. Values that are then recorded in RDSR need to be inserted in SkinCare’s fields *Lateral offset, Longitudinal offset,* and *Height offset*. Additionally, SkinCare also has field *Head‐Table distance* in order to improve the patient position estimation on the table, by taking into account distance from patient head to the table end (this value should be measured prior to the beginning of every procedure). The orientation of the model is assumed to be supine and to lie in the middle of the table.

#### 2.A.3 Skin dose calculation

Skin dose calculation is based on determining the affected points of the 3D patient model by the x‐ray beam. Once the 3D positions of x‐ray tube focal spot, detector, and patient are found for every irradiation event using data from RDSR, dose is calculated as entrance surface dose, that is, only for 3D points in which x‐ray beam enters the patient model (x‐ray beam exit points are not relevant).

Dose is calculated by using [Eq. (1)]:

(1) $$Dose=K_{a,r}\times {CF\times TAF\times F_{\theta }\times \left(\frac{d_{\mathrm{IRP}}}{d_{\mathrm{patient}}}\right)}^{2}\times BSF\times MAEC$$

where $K_{a,r}$ is the air kerma reported at the Interventional Reference Point (IRP),^27^ CF is calibration factor for the KAP meter, TAF is table attenuation factor,$F_{\theta }$ is oblique factor defined as relative fraction of transmission between zero and nonzero angles of incidence, $d_{\mathrm{IRP}}$ is distance from source to IRP, $d_{\mathrm{patient}}$ is distance from source to affected 3D point,BSF is backscatter factor interpolated from the coefficients of Benmakhlouf H *et al*.,^28^ and MAEC is the dose conversion factor from air to soft tissue interpolated from the coefficients of Benmakhlouf H *et al*.^28^

### 2.B SkinCare validation

#### 2.B.1 Gafchromic film calibration

XR‐RV3 Gafchromic films calibration was performed in the Secondary Standard Dosimetry Laboratory of Vinča Institute of Nuclear Sciences. Films were cut in small square pieces of 2 cm × 2 cm and the irradiation was carried out free‐in‐air, by positioning films at 1 m distance from the radiation source. During calibration, film pieces were oriented in such a way that the yellow side of the film was facing the x‐ray tube. The film pieces were irradiated to 16 air kerma values between 0 and 10 Gy using RQR8 standard beam quality.^29^ The conversion of air kerma to absorbed dose to skin is considered to be equal to one.^30^

Scanning of the irradiated films was performed after 24 hr of exposure with the Hewlett‐Packard (HP) Scanjet 7650 flatbed scanner. VueScan scanning software was used for linear (raw) scanning which enables data acquisition straight from the scanner’s sensor without any manipulation from the scanning software. Most software packages that come with consumer scanners do not offer this ability and perform processing on raw data. Film pieces were scanned with a resolution of 300 PPI as 48 Bit RGB TIF files. Scanned images were analyzed in the Python programming language using only the red channel in order to ensure maximum sensitivity. To obtain an image value corresponding to a particular air kerma, a square region of interest (ROI) was formed with dimensions of 120 px (ROI was approximately 1 cm^2^). The formed ROI was shifted throughout the whole image, and pixel values inside the ROI were averaged. ROI with the lowest average pixel value corresponds to the highest dose. Response of the XR‐RV3 Gafchromic films as a function of film dose was modeled using a rational function proposed by Lewis *et al*.^31^:

(2) D=a+b/(x+c)

where x represents the lowest average pixel value of ROI at dose D, and a, b, and c are coefficients of the rational function. Calibration curve was obtained using Levenberg–Marquardt nonlinear curve fitting algorithm.

Uncertainties related to the use of XR‐RV3 Gafchromic films for patient skin dose assessment in the interventional environment have been estimated in our previous work and published in Medical Physics.^30^ It is shown that overall uncertainty of skin dose measurements using XR‐RV3 Gafchromic films ranges from 9% (k = 2) for tightly controlled measurement conditions, adequate laboratory calibrations and well‐defined readout protocol to 78% (k = 2) in the worst case scenario where the conditions of exposure, film handling, and readout are weakly controlled and where corrections for the relevant influence quantities are not made. In this work the overall measurement uncertainty was assumed to be 41% (k = 2) since a well‐defined laboratory calibration is performed, while other influencing parameters related to clinical application of dosimetry films are less controlled.^30^

#### 2.B.2 VERIDIC’s acceptance and QC testing protocols

One of the objectives of the VERIDIC project was to develop a protocol for acceptance and QC tests of SDC software to be used by medical physicists in clinical practice.^24^ A slightly modified acceptance protocol which is originally composed of 13 fundamental irradiation setups and three simplified clinical procedures intended to represent more realistic conditions, has been used. Fundamental setups in addition to simplified clinical procedures are used to verify that key parameters (collimated field area, table height, C‐arm angulation) which can significantly affect the PSD, were appropriately taken into account by SkinCare. All irradiations were performed using fluorographic (acquisition) mode.

First four fundamental irradiations intended to evaluate CF and TAF for two beam qualities were not performed since thorough evaluation of CF and TAF was performed before conducting this protocol in the Siemens service mode over wide range of beam qualities as discussed in the next section. XR‐RV3 Gafchromic films were used as dosimeters to compare PSDs and dose maps obtained through measurements and calculations. For such comparison, a tissue equivalent slab phantom, combined of water and polymethyl methacrylate (PMMA) plates (plates of 1 cm thickness, 30 × 30 cm^2^ surface), was used to simulate a simplified patient of different sizes (10, 15, 20, 25, and 30 cm). Acceptability criteria require that the calculated PSD values should not differ more than 40% from the measured values for any of the fundamental irradiation events and the simplified procedures.^24^

#### 2.B.3 Calibration factor and Table attenuation factor

Two multiplicative factors that significantly influence skin dose calculations are the calibration factor (CF) and the table attenuation factor (TAF). RDSR provides for entry of a single *CF* which corrects air kerma reported at the IRP, $K_{a,r}$, in such a way by calibration of the $K_{a,r}$ against quality control measurements. The allowable tolerance for the displayed $K_{a,r}$ and kerma‐area product (KAP) values should not deviate from the actual values by more than ± 35% above 100 mGy and 2.5 Gy‐cm^2^, respectively.^32^ The effect of the patient table and the mattress on the x‐ray beam transmission is taken into account by using TAF which depends on beam quality, field size, and C‐arm angulation. DeLorenzo *et al*.^33^ found that TAF values at 0° for table plus pad ranged from 0.59 to 0.89 for three different fluoroscopy systems. Such a large tolerance for CF and the fact that TAF is not defined in RDSR could lead to very inaccurate skin dose calculations.

##### Evaluation of calibration factor

**Fig. 1:**
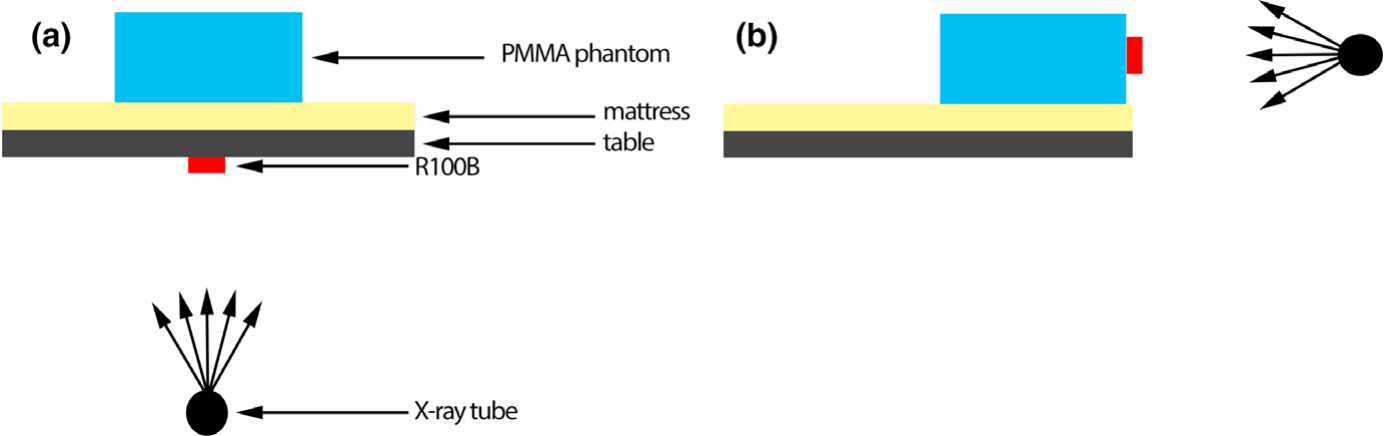
Calibration factor evaluation setup for (a) plane a and (b) plane B. Plane A was set in under‐table position and Plane B was set at the lateral position.

Calibration of the KAP meters in the Siemens Artis Zee Biplane system was performed with a solid‐state semiconductor detector R100B (RTI Electronics AB, Molndal, Sweden), calibrated in the Secondary Standard Dosimetry Laboratory at the Vinča Institute of Nuclear Sciences in standard RQR beam qualities.^29^ Setups used for determination of the calibration factors are shown in Fig 1. The frontal tube (Plane A) was set at the under‐table position (primary angle = 0^ο^, secondary angle = 0^ο^), and the lateral tube (Plane B) was set at the lateral position (primary angle = ±90^ο^, secondary angle = 0^ο^). R100B detector was taped at the beam entrance side of the table for Plane A measurements, while it was taped at the beam entrance side of the PMMA phantom for Plane B measurements. A radiopaque ruler was used to measure dimensions of the x‐ray field which was set to approximately 10 × 10 cm in the plane of the detector.

The Siemens Artis Zee Biplane was operated in service mode in order to determine the CF for different beam qualities. Exposures were made using X‐ray tube voltages ranging from 60 to 120 kVp with 10 kVp increments at six different copper filter thicknesses: 0, 0.1, 0.2, 0.3, 0.6, and 0.9 mm. The KAP measured by the system’s KAP meter and the incident air kerma ($K_{a,i}$) measured by R100B were recorded for each exposure. Calibration factor for each exposure was calculated as:

(3) $$CF=\frac{K_{a,i}\cdot A}{\mathrm{KAP}}$$

where CFis the calibration factor, $K_{a,i}$is the incident air kerma measured by R100B, Ais the field area in the plane of the R100B, and KAPis air kerma‐area product measured by the KAP meter.

##### Evaluation of table attenuation factor

**Fig. 2:**
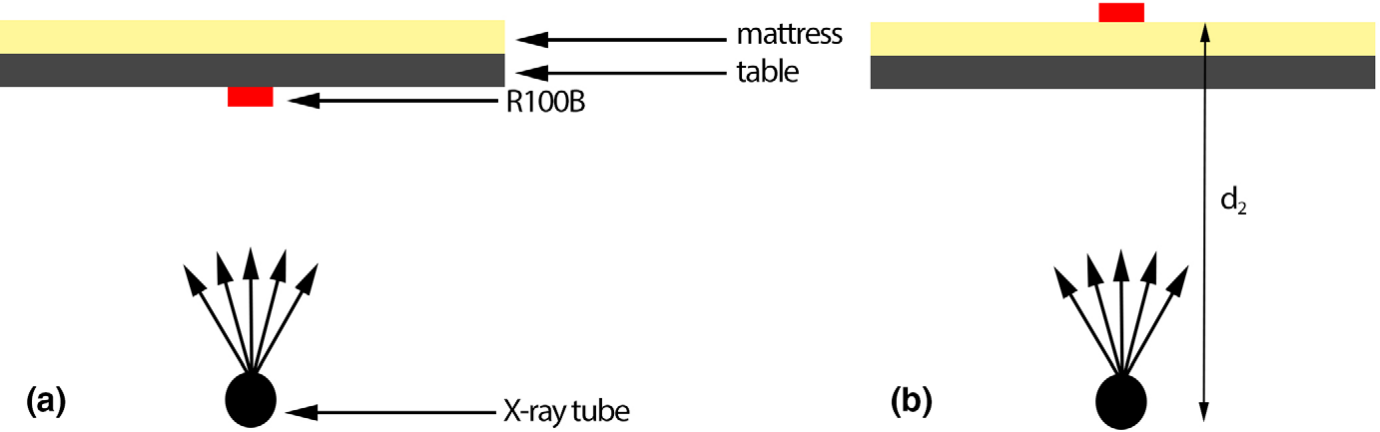
Table attenuation factor measurements for (a) setup 1 and (b) setup 2. Setup 1 was used to measure air kerma rates when there was no influence of table, whereas setup 2 was used to measure air kerma rates when table and mattress were in the beam path.

TAF was measured using the frontal tube (Plane A) set at the under‐table x‐ray tube position (primary angle = 0^ο^, secondary angle = 0^ο^) using detector R100B. Setups used for determination of TAF values are shown in Fig 2. Setup 1 was used to measure air kerma rates without the patient table influence, while setup 2 was used to measure air kerma rates when table and mattress were in the beam path. A radiopaque ruler was used to measure dimensions of x‐ray field for setup 2.

Service‐mode projections were acquired for each value of the x‐ray tube voltage ranging from 60 to 120 kVp with 10 kVp increments and varying copper thicknesses of 0, 0.1, 0.2, 0.3, 0.6, and 0.9 mm. Also, three field sizes of 10 × 10 cm^2^, 15 × 15 cm^2^, and 20 × 20 cm^2^ in the plane of R100B have been employed. Air kerma rates measured by R100B were recorded for each exposure. Table attenuation factor for each exposure was calculated as:

(4) $$TAF=\frac{K_{a,2}}{K_{a,1}\cdot {\left(\frac{d_{1}}{d_{2}}\right)}^{2}}$$

where TAFis the table attenuation factor, $K_{a,1}$is the air kerma rate from setup 1,$K_{a,2}$ is the air kerma rate from setup 2, and the ratio ${\left(\frac{d_{2}}{d_{1}}\right)}^{2}$stands for the inverse‐square‐law correction for distance.

For a beam incident on the table surface at a nonperpendicular angle, θ, TAF should be multiplied with oblique factor, $F_{\theta }$, which is defined as a relative fraction of transmission between zero and nonzero angles of incidence. Using methodology proposed by Rana et al.,^9^ oblique factor was calculated as:

(5) $$F_{\theta }=e^{\log\left(TAF\right)\cdot \left(\sec\left(\theta \right)‐1\right)}$$

#### 2.B.4 Comparison of patient skin dose maps generated using different commercial software tools

Apart from acceptance protocol, the accuracy of SkinCare was evaluated by visual comparison of skin dose maps generated by SkinCare and different commercial software tools using DICOM RDSRs generated after cardiology clinical procedures (percutaneous coronary intervention‐PCI) performed on patients. These procedures were performed on fluoroscopy units from different vendors (Canon, GE, Philips and Siemens) and their corresponding RDSRs were used to generate skin dose maps by SkinCare and to compare them to skin dose maps generated by different commercial software tools (DTS from Canon, RadimetricsTM from Bayer and RDM from MEDSQUARE). These skin dose mapping software tools were chosen because they have been validated against the fluoroscopy units from above‐mentioned manufacturers. The main objective of this comparison was to verify the correctness of applied geometric algorithm for different manufacturers, while PSD comparison was not performed because the lack of the knowledge of fluoroscopy system specific values of CF and TAF would result in incorrect comparison.

## 3 RESULTS

### 3.A SkinCare

**Fig. 3:**
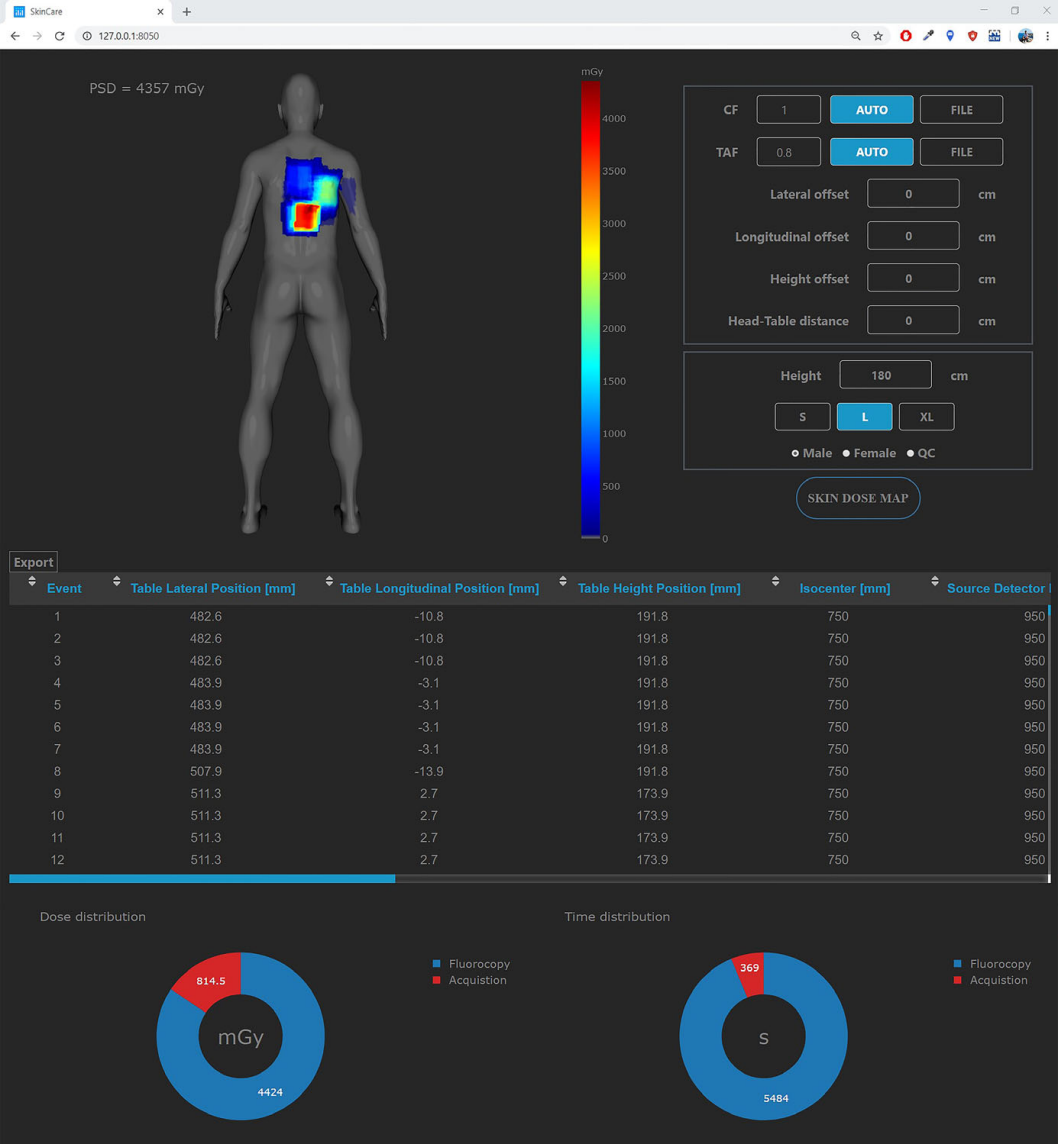
SkinCare user interface.

The graphical user interface (GUI) of SkinCare is shown in Figure 3. It is a single‐page long‐scrolling standalone application that runs in any web browser. Modern design and ease of use enables very fast acquisition of skin dose map and PSD.

SkinCare enables usage of single CF and TAF values through the *AUTO* mode (by pressing AUTO button) or multiple CF and TAF values via the *FILE* mode (by pressing *FILE* button). In the *FILE* mode user needs to choose a .csv file filled with values for given template. Table 1 and Table 2 present templates filled with arbitrary values for CF and TAF determination, respectively. In Table 2 “FOV” corresponds to table top square field size. There is no limit in number of rows for templates, so it is up to medical physicists to decide on number of measurements. Last two columns of templates correspond to CF and TAF values for fluoroscopy and image acquisition, thus this mode enables very accurate skin dose assessment. In this work we only acquired CF and TAF for image acquisition but it is possible to acquire separate factors for both fluoroscopy and image acquisition. Interpolated CF and TAF values are then calculated for every irradiation event based on values inserted in templates.

**Table 1:** Template for CF determination.

kV [kV]	Cu [mm]	CF fluoroscopy	CF image acquisition
60	0	0.80	0.80
80	0.1	0.81	0.81
100	0.2	0.82	0.82
120 ⋮	0.3 ⋮	0.83 ⋮	0.83 ⋮

**Table 2:** Template for TAF determination.

FOV [cm]	kV [kV]	Cu [mm]	TAF fluoroscopy	TAF image acquisition
7	60	0	0.80	0.80
7	80	0.1	0.81	0.81
7	100	0.2	0.82	0.82
7 ⋮	120 ⋮	0.3 ⋮	0.83 ⋮	0.83 ⋮

Patient model configuration is performed by setting the height, size, and gender of a patient. Apart from patient models, there are two additional models (slab phantom and XR‐RV3 Gafchromic film) which enable medical physicists to perform QC test of the system. Rotation of the models enables easier visualization of the skin dose map, especially when larger angulations and oblique projections are present.

Below the skin dose map is scrollable table in horizontal and vertical directions which contains data from RDSR (event number, table lateral position, table longitudinal position, table height position, source to isocenter distance, source to detector distance, event type, primary angle, secondary angle, air kerma at IRP, tube voltage, and copper filtration) and data calculated by SkinCare (field‐of‐view, BSF, CF, TAF, field‐of‐view at entrance side of patient, and source to patient distance) for each irradiation event. Two doughnut charts that show image acquisition vs fluoroscopy dose and time distributions are positioned below the table.

**Fig. 4:**
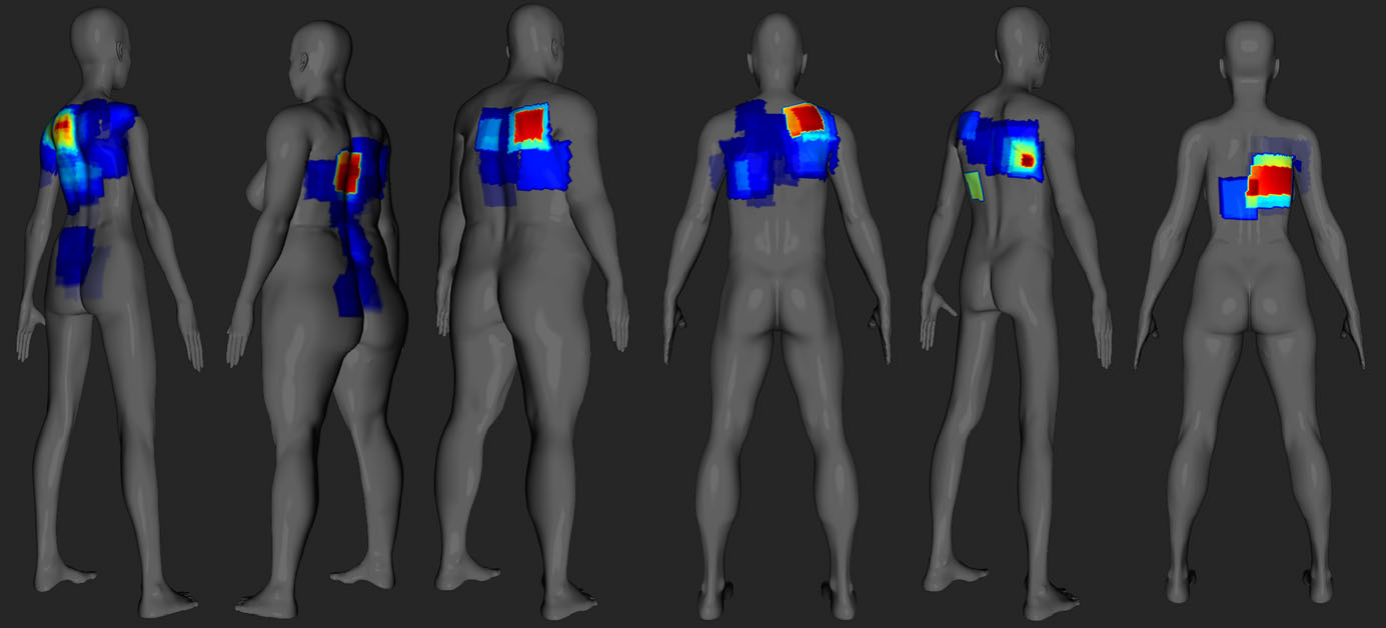
Skin dose maps for different RDSRs. Color map is relative to the PSD for each individual patient.

Skin dose maps for different RDSRs are shown in Fig 4. Color coded dose map is relative to the PSD for each individual patient. Figure 4 highlights the effectiveness of SkinCare for producing very detailed dose maps from different fluoroscopy system manufacturers on a diverse patient population.

**Fig. 5:**
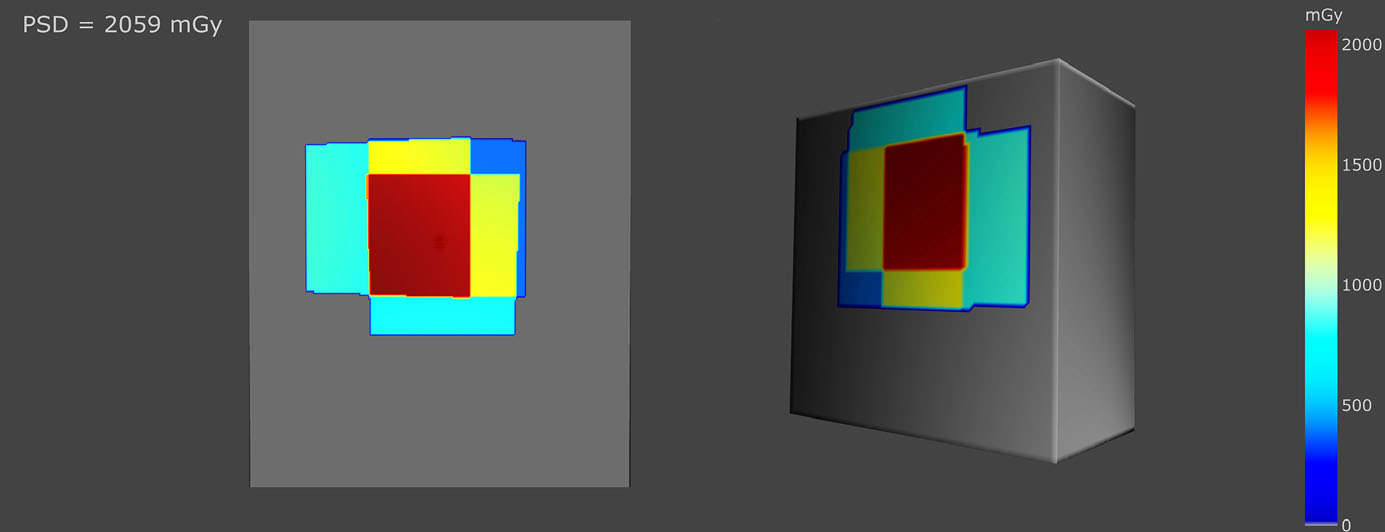
Models of XR‐RV3 Gafchromic film (left) and slab phantom (right) with dose maps.

Figure 5 shows models of the XR‐RV3 Gafchromic film (left) and slab phantom (right). Since the models have flat surfaces and good spatial resolution, medical physicists can perform fast measurements for the purpose of QC tests.

### 3.B SkinCare validation

#### 3.B.1 Gafchromic film calibration

**Fig. 6:**
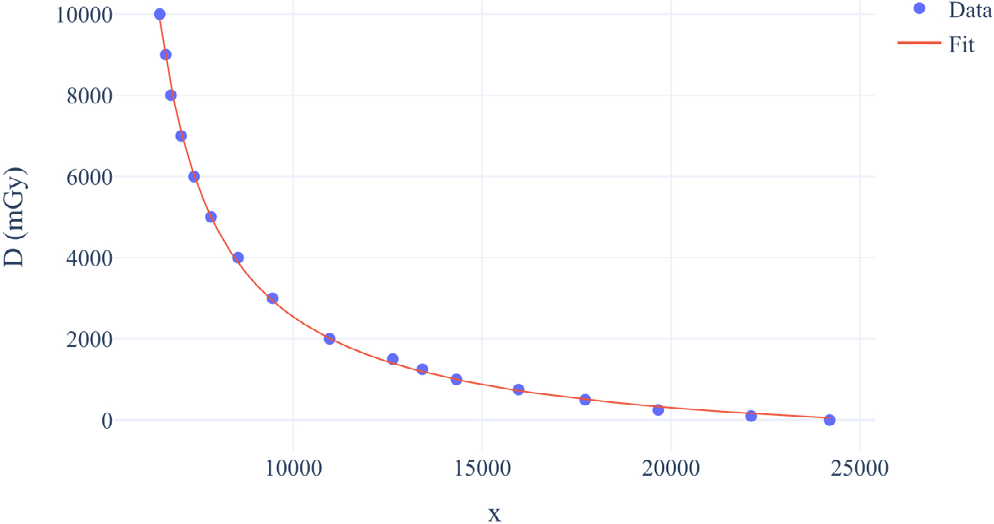
XR‐RV3 Gafchromic calibration curve.

XR‐RV3 Gafchromic calibration curve for determining skin dose is shown in Figure 6. Fitting coefficients have the following values: $a=‐851.1,b=1.754*10^{7},c=‐4828$. Table 3 shows measured values, predicted values, and percent errors for all data used during calibration. Fitting errors in the high dose range (>2 Gy) where tissue reactions may occur to the skin are within 3.2%. Additionally, root mean square error (RMSE) goodness‐of‐fit indicator was calculated in order to evaluate the performance of the fitting equation. Fitting equation produces an RMSE of 92 mGy which demonstrates small error of fitting.

**Table 3:** Assessment of fitting function from Equation 2.

Point	Measured (mGy)	Predicted (mGy)	Error (%)
1	100	162.6	62.6
2	250	330.7	32.28
3	500	508.1	1.62
4	750	723.4	−3.55
5	1000	996.4	−0.36
6	1250	1190.4	−4.77
7	1500	1395.5	−6.97
8	2000	2007.8	0.39
9	3000	2942.5	−1.92
10	4000	3875.1	−3.12
11	5000	5007.5	0.15
12	6000	6040.0	0.66
13	7000	7110.3	1.57
14	8000	8228.8	2.86
15	9000	8910.1	−1.00
16	10000	9867.0	−1.33

#### 3.B.2 Calibration factor and Table attenuation factor

**Fig. 7:**
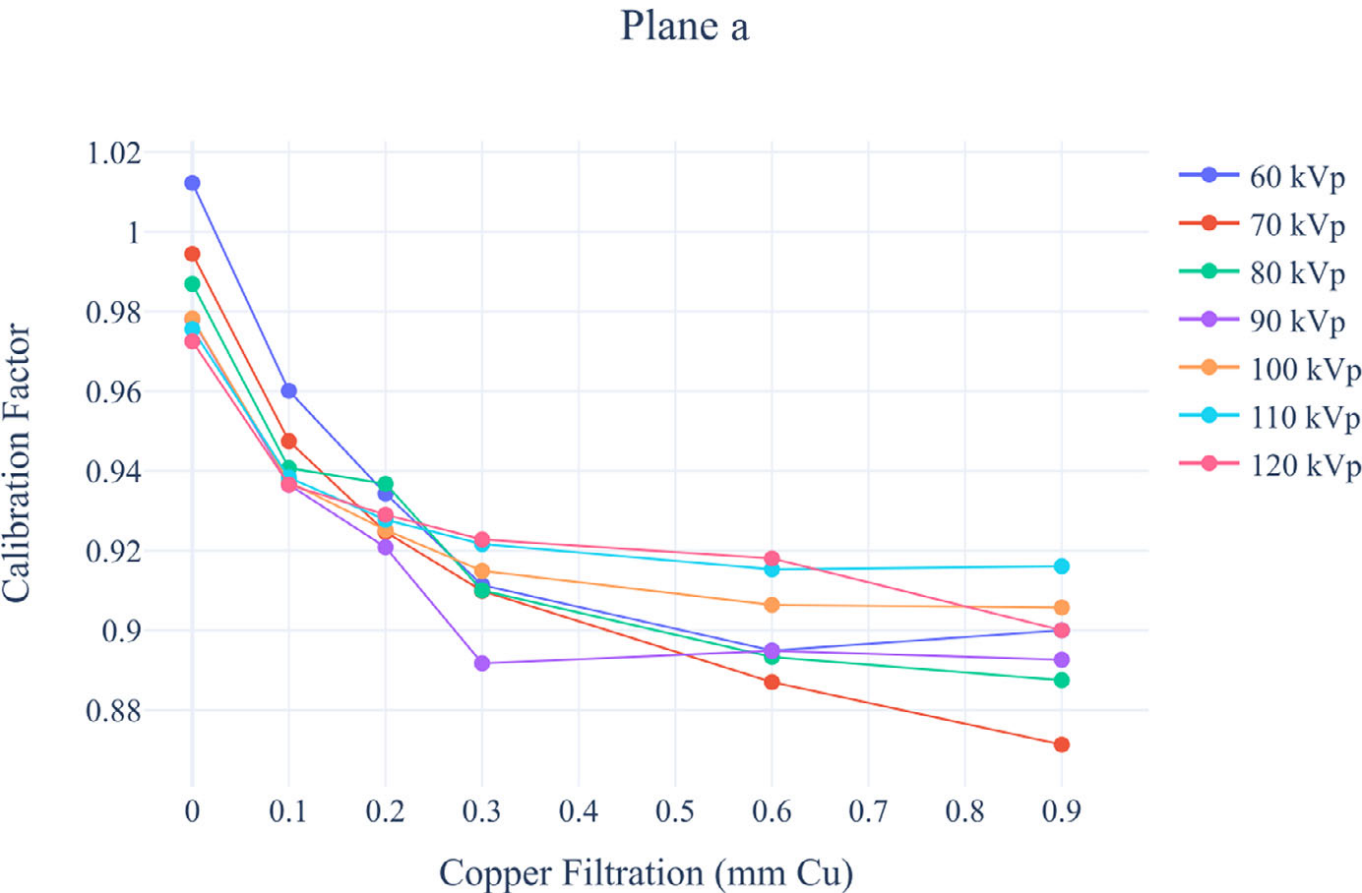
Siemens Artis Zee Biplane fluoroscopy system calibration factors for Plane a.

**Fig. 8:**
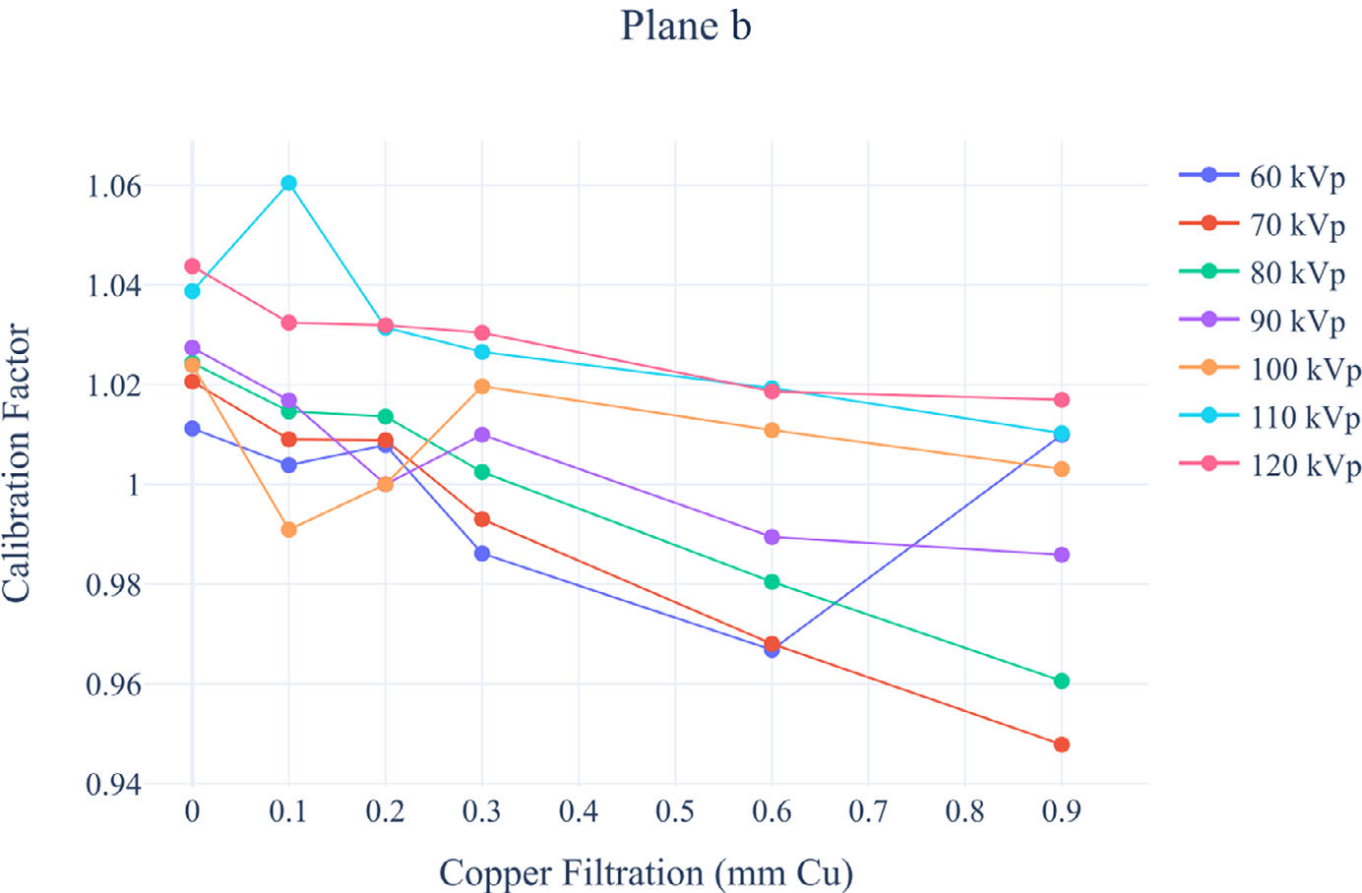
Siemens Artis Zee Biplane fluoroscopy system calibration factors for Plane b.

Calibration factors for each beam quality for both tubes of biplane system are presented in Figures 7 and 8. For each particular filter thickness for all tube voltages, differences between the lowest and the highest calibration factors values were calculated. For Plane A differences ranged from 1.3% to 4.5%, whereas for Plane B differences ranged from 3.2% to 7.0%.

Table attenuation factors for Siemens Artis Zee Biplane fluoroscopy system can be found in Table 4. With the increase in the x‐ray tube voltage and by increasing the field size and added Cu filtration of the primary x‐ray beam, TAF value has increased. The recorded TAF values ranged from 0.69 to 0.88. Table 5 shows the oblique factor $F_{\theta }$ as a function of incident angle, kVp and added Cu filtration for the 10 x 10 cm^2^ field size. $F_{\theta }$ decreased with the increase in the incident angle and at 10^ο^, 20^ο^, 30^ο^, and 40^ο^ ranged from 0.994 to 0.997, 0.977 to 0.989, 0.945 to 0.973, and 0.894 to 0.948, respectively. Field size had a small influence on $F_{\theta }$.

**Table 4:** Siemens Artis Zee Biplane fluoroscopy system table attenuation factors.

Field Size (cm^2^)	Tube voltage (kV)	Additional filtration (mm Cu)					
		0	0.1	0.2	0.3	0.6	0.9
10 × 10	60	0.694	0.745	0.769	0.783	0.808	0.825
	70	0.713	0.762	0.785	0.798	0.822	0.833
	80	0.724	0.772	0.793	0.806	0.827	0.836
	90	0.734	0.779	0.799	0.810	0.829	0.836
	100 110	0.743	0.785	0.805	0.814	0.831	0.836
		0.750	0.790	0.808	0.817	0.832	0.838
	120	0.756	0.794	0.811	0.823	0.834	0.839
15 × 15	60	0.703	0.755	0.782	0.796	0.823	0.834
	70	0.721	0.774	0.798	0.810	0.838	0.848
	80	0.732	0.783	0.807	0.819	0.842	0.851
	90	0.743	0.790	0.812	0.823	0.845	0.848
	100	0.751	0.796	0.817	0.827	0.845	0.851
	110	0.759	0.801	0.821	0.830	0.845	0.852
	120	0.765	0.805	0.824	0.833	0.848	0.853
20 × 20	60	0.720	0.772	0.795	0.814	0.830	0.843
	70	0.741	0.791	0.812	0.830	0.849	0.860
	80	0.753	0.802	0.823	0.840	0.856	0.866
	90	0.765	0.811	0.830	0.847	0.862	0.870
	100	0.775	0.817	0.839	0.851	0.866	0.874
	110	0.784	0.823	0.844	0.855	0.867	0.876
	120	0.790	0.828	0.849	0.858	0.871	0.878

**Table 5:** Siemens Artis Zee Biplane table oblique factors.

Field Size (cm^2^)	Tube voltage (kV)	Additional filtration (mm Cu)	10^ο^	20^ο^	30^ο^	40^ο^
10 × 10	60‐120	0	0.994‐0.996	0.977‐0.982	0.945‐0.958	0.894‐0.918
	60‐120	0.1	0.995‐0.996	0.981‐0.985	0.955‐0.965	0.914‐0.932
	60‐120	0.2	0.996‐0.997	0.983‐0.987	0.960‐0.968	0.923‐0.938
	60‐120	0.3	0.996‐0.997	0.984‐0.988	0.963‐0.970	0.928‐0.942
	60‐120	0.6	0.997‐0.997	0.986‐0.988	0.968‐0.972	0.937‐0.946
	60‐120	0.9	0.997‐0.997	0.988‐0.989	0.971‐0.973	0.943‐0.948

#### 3.B.3 VERIDIC’s acceptance and QC testing protocols

In Table 6 eight fundamental irradiation setups used to validate SkinCare and PSD comparison between XR‐RV3 Gafchromic films and SkinCare are displayed. During all irradiations the tube voltage and the added filtration were automatically set by the system, while RDSR attributes *Table Height position*, *Distance source to detector,* and *Collimated field area* were set at 15 cm, 120 cm, and 900 cm^2^, respectively. First five setups were used for the purpose of testing the effect of the phantom scatter, sixth and seventh were used for testing of the effect of field overlap, whereas eighth was used for testing the effect of lateral irradiations. The tests have shown that PSD calculated by SkinCare is within 17% accuracy compared with measurements using XR‐RV3 Gafchromic films.

**Table 6:** Fundamental irradiation setups.

Setup	Configuration	Tube projection	Dose (IRP) total (mGy)	PSD (mGy)		Accuracy
				XR‐RV3	SkinCare	
1*	Phantom 15 cm + table + mattress	PA	968	1362	1184	−13.1%
2*	Phantom 20 cm + table + mattress	PA	673	784	765	−2.4%
3*	Phantom 25 cm + table + mattress	PA	556	605	630	4.1%
4*	Phantom 30 cm + table + mattress	PA	1180	1463	1414	−3.3%
5*	Phantom 10 cm + table + mattress	PA	96	130	108	−16.9%
6*	Phantom 25 cm + mattress + table	PA + LAO 20^ο^	1528	2187	1947	−11.0%
7*	Phantom 25 cm + mattress + table	PA + LAO 20^ο^ + PA CRAN 15^ο^	1638	2240	2086	−6.9%
8*	Phantom 25 cm + mattress + table	LAO 90^ο^	1317	1777	1869	5.2%

* Table Height position = 15 cm, Distance source to detector = 120 cm, FOV = 29 cm, tube voltage and additional filtration (mm Cu) ‐ Automated selection.

**Fig. 9:**
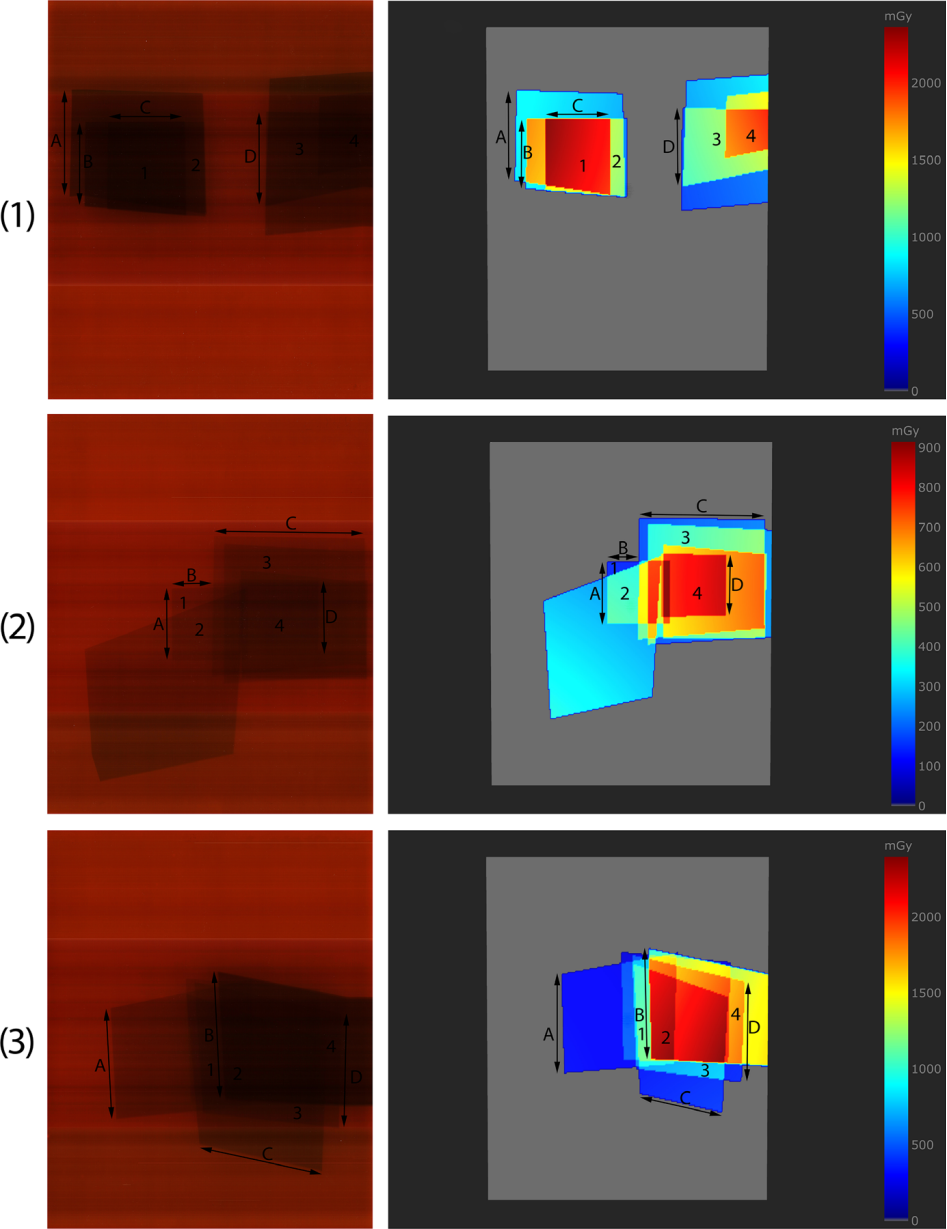
Comparison of the dose maps obtained using XR‐RV3 Gafchromic films (left) and SkinCare’s XR‐RV3 Gafchromic phantom (right) for three simplified clinical procedures. On both images for the same procedures there are letters a‐d used for comparison of the spatial distribution of dose and numbers 1–4 used for dose comparison. First row represents first procedure, second row second procedure, and third row third procedure.

Table 7 shows setups of the simplified clinical procedures. During all irradiations the tube voltage and the added filtration were automatically set by the system, while the distance source to detector was kept constant at 120 cm. Slab phantom with an addition of 10 cm PMMA layer were used as the phantom. Procedures varied considerably as it can be seen from Table 7 and Fig. 9. Figure 9 shows comparison between scanned XR‐RV3 Gafchromic films and dose maps obtained using SkinCare’s XR‐RV3 Gafchromic phantom for all three simplified clinical procedures. It should be noted that the scanner used for geometrical comparison in Figure 9 was not used for calibration and PSD assessment due to the presence of horizontal lines on scanned images. On both of the images for the same procedures there are letters A–D used for comparison of the spatial distribution of dose and numbers 1–4 used for dose comparison. In Table 8 the comparison results are displayed. The doses calculated by SkinCare were within 16% accuracy compared with measurements using XR‐RV3 Gafchromic films. Spatial distribution of dose as calculated on the SkinCare’s XR‐RV3 Gafchromic phantom were within 9% accuracy compared to spatial distributions recorded on the GafChromic film for all points except for one point where accuracy was −20.7% (2.8 cm in this case).

**Table 7:** Simplified clinical procedures.

Procedure	Primary Angle (degrees)	Secondary Angle (degrees)	Collimated Field Area (cm^2^)	Table Height Position (cm)	Cumulative K_a,r_ (mGy)
1*	−30	−10	745	15	498
	−30	−5	439	15	533
	−25	−5	439	15	431
	30	−15	751	15	469
	30	−5	751	15	346
	35	−15	285	15	372
2*	30	5	506	10	191
	25	−5	815	10	176
	30	0	815	10	183
	−10	5	234	10	141
	20	0	234	10	165
	−30	30	838	10	221
3*	20	10	552	5	248
	50	10	552	5	461
	−30	15	552	5	277
	25	35	552	5	327
	30	20	552	5	320
	50	10	552	5	703

* Distance source to detector = 120 cm, tube voltage and additional filtration (mm Cu) ‐ Automated selection.

**Table 8:** Comparison of the doses and spatial distribution of dose measured by XR‐RV3 Gafchromic films and SkinCare.

Procedure	Point	Dose (mGy)		Accuracy	Point	Distance (cm)		Accuracy
		XR‐RV3	SkinCare			XR‐RV3	SkinCare	
1	1	1794	1809	0.8%	A	11.4	11.4	0.0%
	2	1042	1078	3.5%	B	9.0	8.9	−1.1%
	3	1068	1025	−4.0%	C	8.2	8.2	0.0%
	4	1505	1552	3.1%	D	10.1	9.9	−2.0%
2	1	127	133	4.7%	A	7.5	8.0	6.7%
	2	310	358	15.5%	B	4.4	4.0	−8.6%
	3	422	370	−12.3%	C	16.0	16.0	0.0%
	4	781	721	−7.7%	D	7.4	7.5	1.1%
3	1	1079	1015	−5.9%	A	11.9	12.5	5.0%
	2	1898	1971	3.8%	B	13.8	14.1	2.2%
	3	565	647	14.5%	C	13.5	10.7	−20.7%
	4	1556	1464	−5.9%	D	12.4	12.6	1.6%

#### 3.B.4 Comparison of patient skin dose maps generated using different commercial software tools

**Fig. 10:**
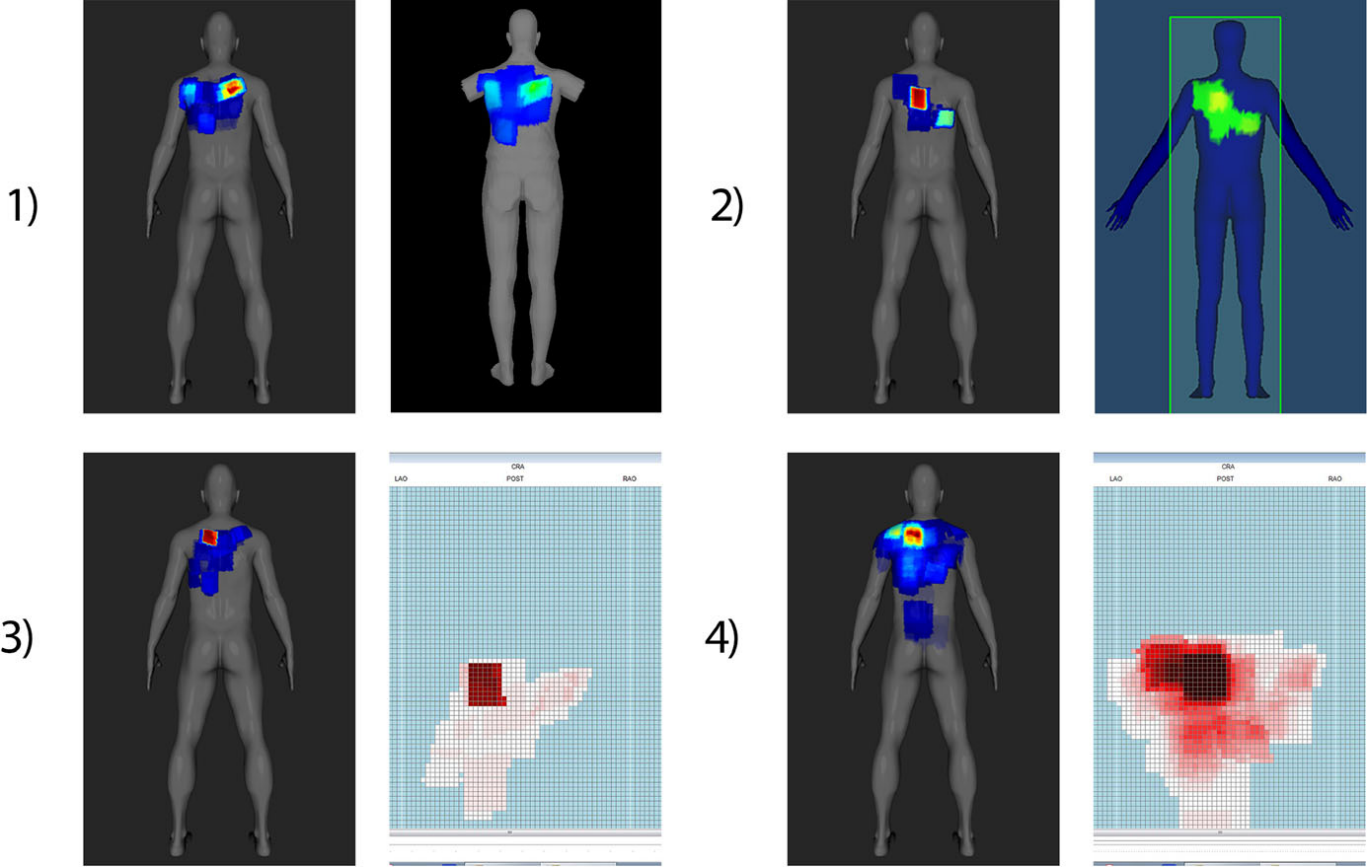
Dose map comparison between SkinCare and 1) DTS for Canon RDSR, 2) Radimetrics^TM^ for Philips RDSR, 3) RDM for GE RDSR, 4) RDM for Siemens RDSR.

Figure 10 presents comparison of a skin dose map generated by SkinCare to skin dose maps from different commercial software tools, all based on the same RDSRs generated during routine clinical PCI procedure. In particular, RDRSs from Canon, Philips, and GE/Siemens fluoroscopy units were used to compare skin dose maps from SkinCare to skin dose map from DTS, RadimetricsTM, and RDM, respectively. Good visual agreement between dose maps proved that SkinCare’s geometry methodology is correct for all major vendors.

From the presented results, it is evident that 3D patient representation clearly shows advantages compared to 2D patient representation, especially in the case of larger angulations and oblique projections. Unlike other software which display color maps relative to the PSD, RadimetricsTM displays color map on an absolute dose scale which makes harder to locate highest irradiated region on patient’s skin. Furthermore, RadimetricsTM has slightly worse spatial resolution of dose maps, which makes edges of dose maps sawtooth‐shaped. In addition, DTS models without arms are good way to address cases when patient’s arms are in overhead position, unlike RadimetricsTM and SkinCare which calculate skin dose for patient’s arms if they are in x‐ray beam path.

## 4 DISCUSSION

Standalone vendor‐independent application SkinCare presented in this paper is elegant and efficient way for skin dose mapping in interventional radiology and interventional cardiology. Validation performed by using the data from the Siemens Artis Zee Biplane fluoroscopy system and conducting VERIDIC’s “Acceptance and quality control protocols for skin dose calculating software solutions in interventional cardiology” showed that PSD calculated by SkinCare was within 17% accuracy compared with measurements using XR‐RV3 Gafchromic films for fundamental irradiation setups, whereas doses calculated by SkinCare were within 16% accuracy compared with measurements using XR‐RV3 Gafchromic films for simplified clinical procedures. With respect to the overall uncertainty of skin dose measurements using Gafchromic films, that ranges from 9% to 78%, depending on the level of control of various film dosimetry steps,^30^ the difference of < 20% is considered to be acceptable.

The obtained results are in the same range as those obtained with similar studies that were carried out by other research groups by evaluating software with predefined protocol. Bordier et al.^5^ compared a calculation method that produces dose maps using Gafchromic XR‐RV3 films on an anthropomorphic phantom and found that doses agreed within better than 15% compared with the Gafchromic films. Using the Radiation Dose Monitor tool, Habib Geryes et al.^3^ accomplished average difference of 10 ± 7% and 9 ± 7% between the calculated and the measured PSD values for 34 test conditions performed on PMMA phantom using Siemens Artis Zee and GE Innova IGS interventional systems, respectively. Methodology to evaluate software described by Habib Geryes et al. was used in study by Greffier et al.^18^ and it was found that average differences between the measured PSD by XR‐RV3 Gafchromic films and the calculated PSD using interactive Skin Dose Map® tool (SDMTool) integrated to the radiation dose management system (RDMS) DoseWatch® were 6% ± 6% (range from − 3% to 22%) for flat phantom and 5% ± 7% (range from − 3% to 25%) for ICRP phantom. Rana et al.^9^ found that biplane dose tracking system (Biplane‐DTS) was able to determine the entrance dose within 6% and the spatial distribution of the dose within 4% compared to the measurements with the ionization chamber and film for the SK150 head phantom.

Quantitative analysis by comparing geometry of dose maps between Skincare and other commercial software was not possible to conduct due to lack of information about input parameters for the calibration and table offsets used by other solutions. Nevertheless, good visual agreement between dose maps obtained by SkinCare and commercial software tools (DTS from Canon, Radimetrics^TM^ from Bayer and RDM from MEDSQUARE) proved that SkinCare can be used by medical physicists in practice for most of the modern fluoroscopy units.

Two multiplicative factors that significantly influence skin dose calculations are CF and TAF. RDSR have only one CF, whereas TAF is not defined in RDSR. Additionally, a large range of possible TAF values and allowable tolerance of ± 35% above 100 mGy and 2.5 Gy‐cm^2^ for the displayed $K_{a,r}$ and KAP, respectively, could lead to very inaccurate skin dose calculations. Wunderle *et al*.^34^ showed that for typical adult beam qualities, applying a single CF determined at tube voltage of 100 kV with cooper filtration in the beam results in a deviation of less than 5% due to beam quality variation. Therefore, attribute in RDSR containing multiple values for these factors is mandatory since single CF and unavailable TAF cannot account for typical beam qualities used during interventional procedures.

SkinCare calculates skin dose map and PSD completely based on RDSR, thus any difference between RDSRs from different vendors greatly affect calculation. For instance, only Siemens and Philips have defined *Table Lateral Position* and *Table Longitudinal Position* fields according to the DICOM standard, however, some RDSRs from Philips record these fields in RDSR only for cine acquisitions. Similarly, Siemens and GE report square fields by automatically converting rectangular fields into square equivalent fields which can affect spatial distribution of dose as shown in Table 8, whereas Toshiba’s (Canon) RDSR fields *Collimated field height* and *Collimated field width* when multiplied give a result which is larger than the value in *Collimated field area*. Philips has defined fields *Bottom shutter, Left shutter, Right shutter, and Top shutter* at 1 m from the focal spot in some DICOM conformance statements while in others definition is not provided so it is up to medical physicist to check this in practice. GE sometimes does not report additional filtration while Philips in some RDSRs does not report *Distance Source to Detector* for every event.

From above it is obvious that taking into account all possible vendor specific exceptions can be a problem. Thus, harmonizing RDSRs is mandatory for easier and more accurate assessment of PSD. Additionally, if vendors come up with accurate way of assessing patient position on table and implement that in RDSR, then cumulative skin dose could be accurately calculated for repeated procedures.

## 5 CONCLUSION

This paper presents originally developed standalone desktop application SkinCare for generating skin dose maps and PSD calculation after completion of interventional radiology and cardiology procedures on realistic 3D patient models using DICOM RDSRs from Canon (Toshiba), GE, Philips, and Siemens fluoroscopy systems. SkinCare is proved to be very convenient solution that can be used for monitoring delivered dose following the interventional procedures. The goal of future research is the validation considering the clinical use of SkinCare and expanding SkinCare’s capabilities to support all interventional radiology and cardiology procedures and setups.

## CONFLICT OF INTEREST

The authors have no relevant conflict of interest to disclose.

## Author Contribution

Marko Krajinović and Olivera Ciraj‐Bjelac conceived of the presented idea. Marko Krajinović developed the software and performed the computations. Nikola Kržanović performed Gafchromic film calibration, R100B calibration, and performed calibration curve fitting. Olivera Ciraj‐Bjelac encouraged Marko Krajinović to evaluate Calibration factors and Table attenuation factors in the Siemens service mode over wide range of beam qualities. Olivera Ciraj‐Bjelac supervised the findings of this work. All authors discussed the results and contributed to the final manuscript.
